# Supplementary figures and images for: Apis mellifera propolis enhances apoptosis and invasion inhibition in head and neck cancer cells
Source: PeerJ. 2021 Sep 8;9:e12139. doi: 10.7717/peerj.12139 (PMC8434809; doi:10.7717/peerj.12139)

# Generic Display Report (all)

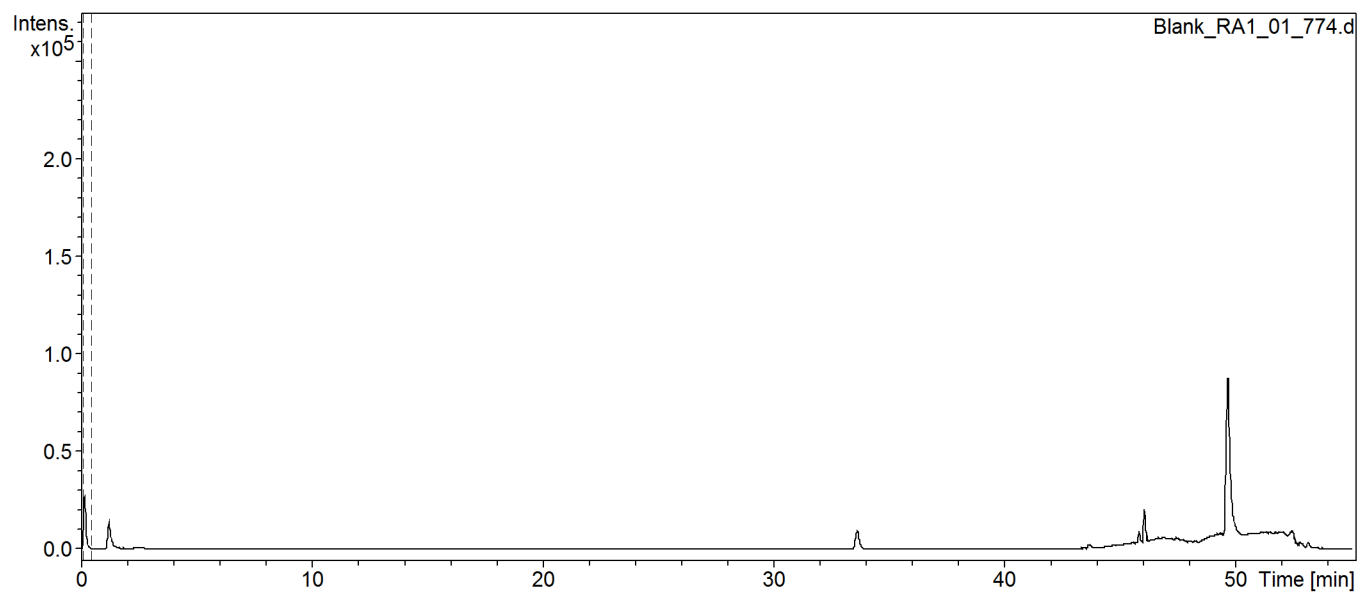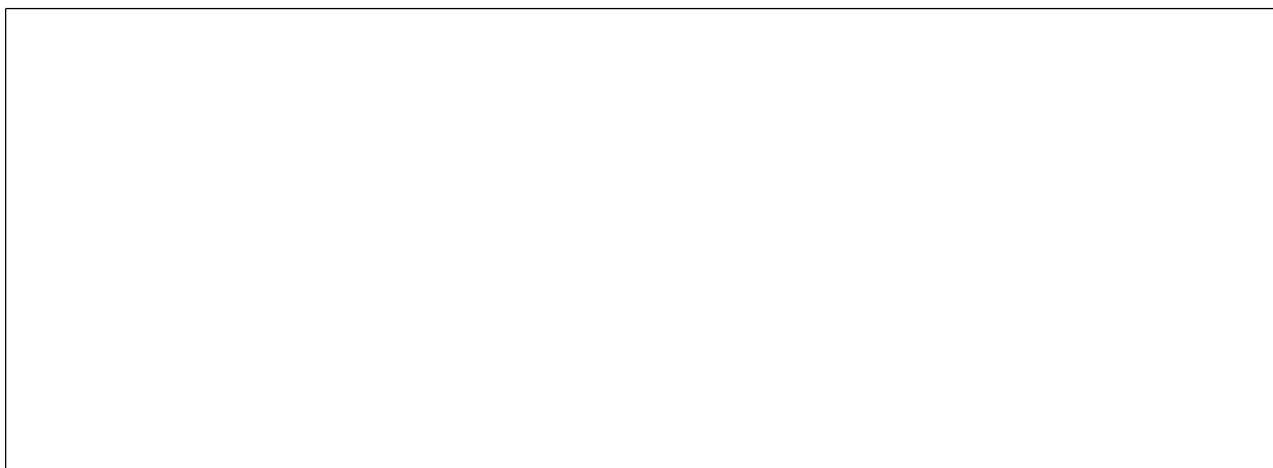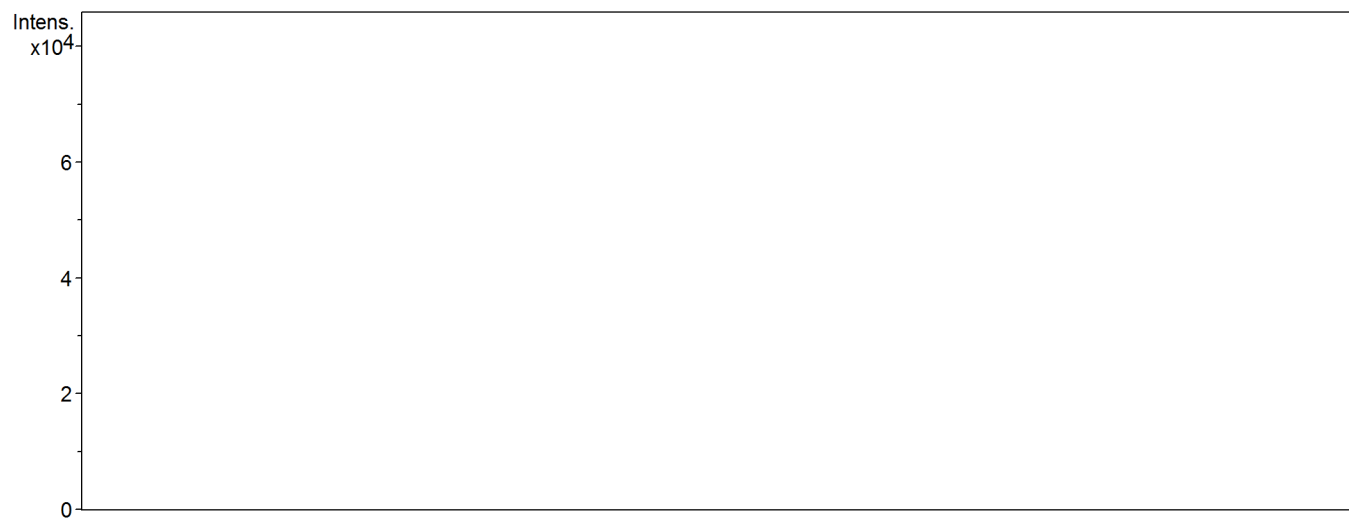

Supplement: Supplemental Information 1 [file peerj-09-12139-s001.pdf]

# Generic Display Report (all)

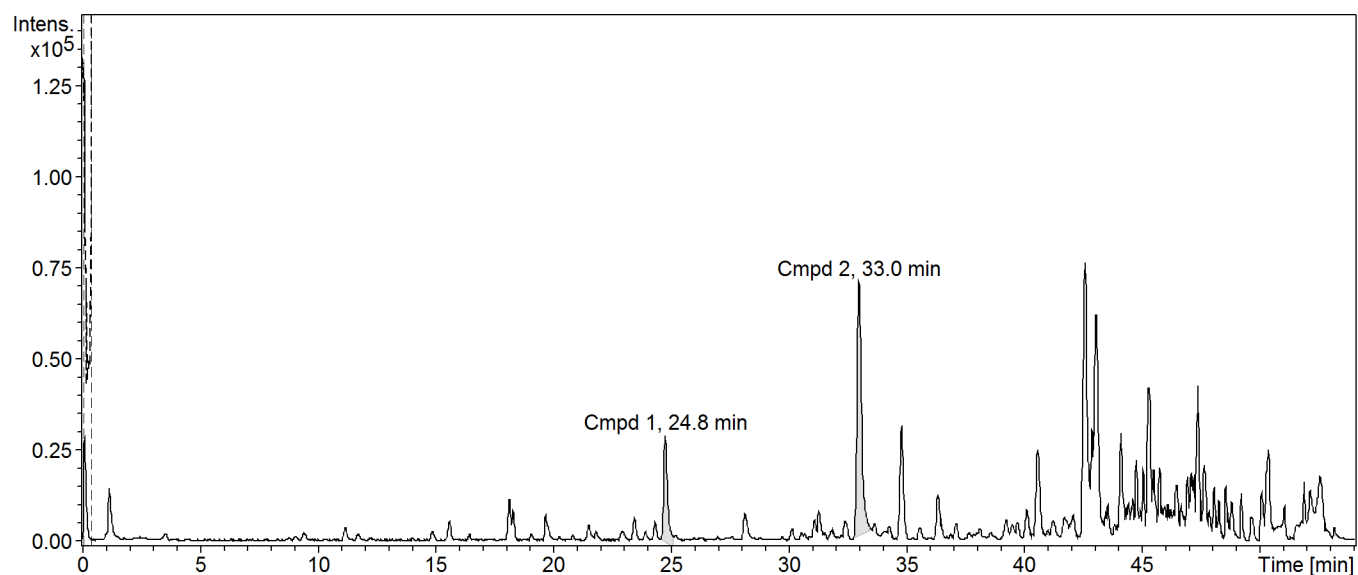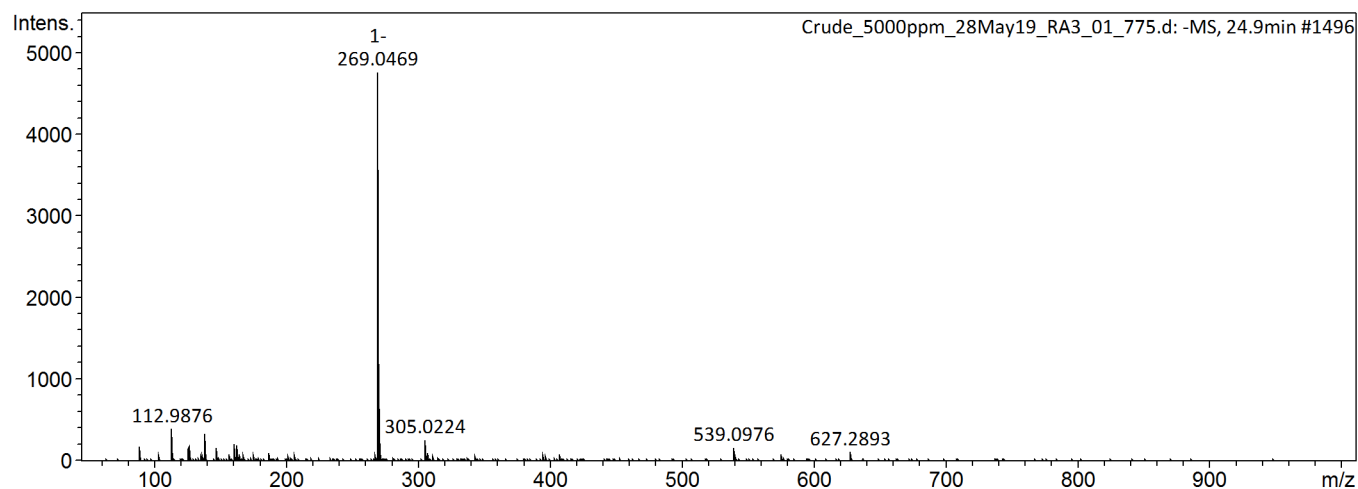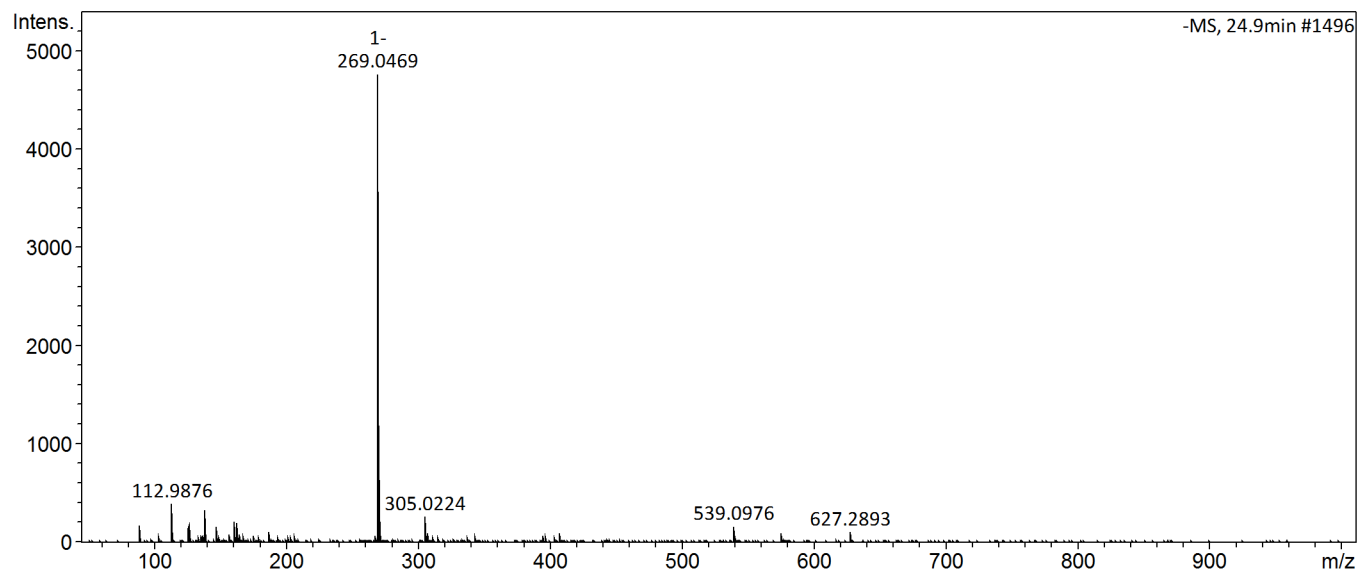

Supplement: Supplemental Information 2 [file peerj-09-12139-s002.pdf]

# Generic Display Report (all)

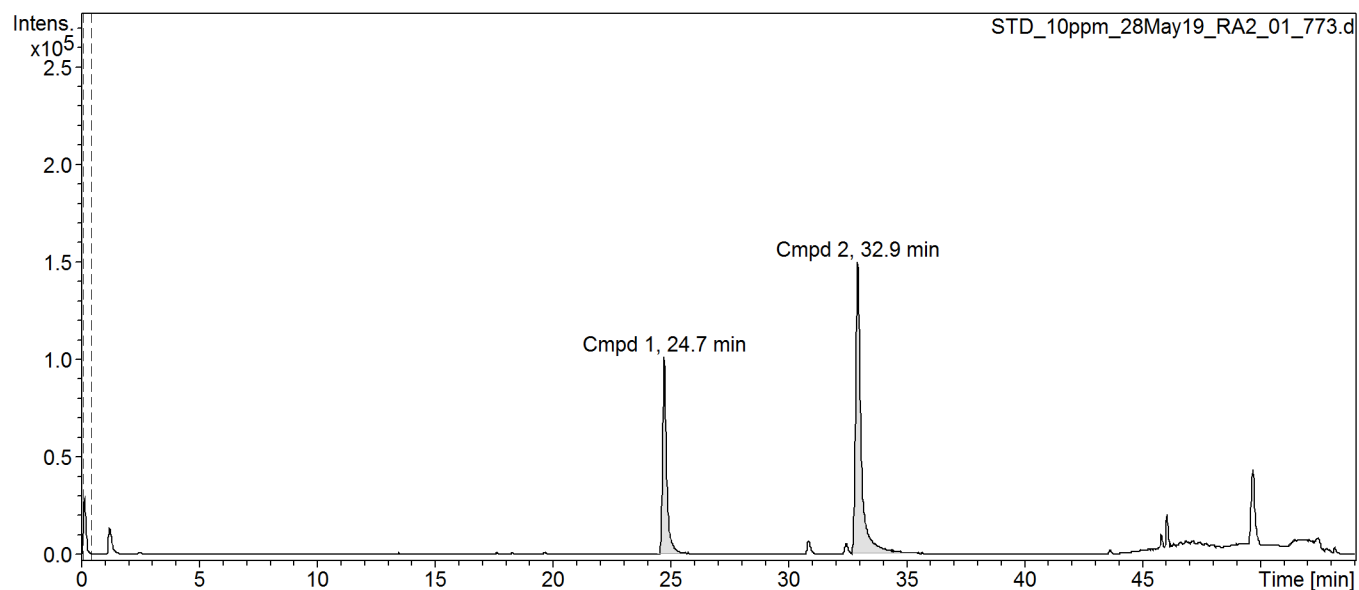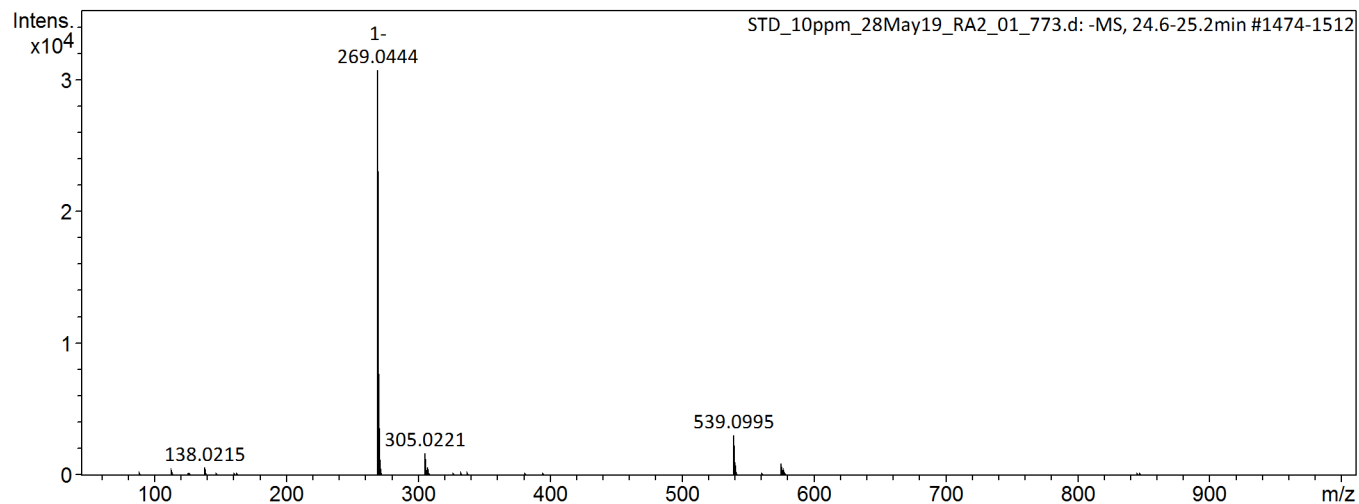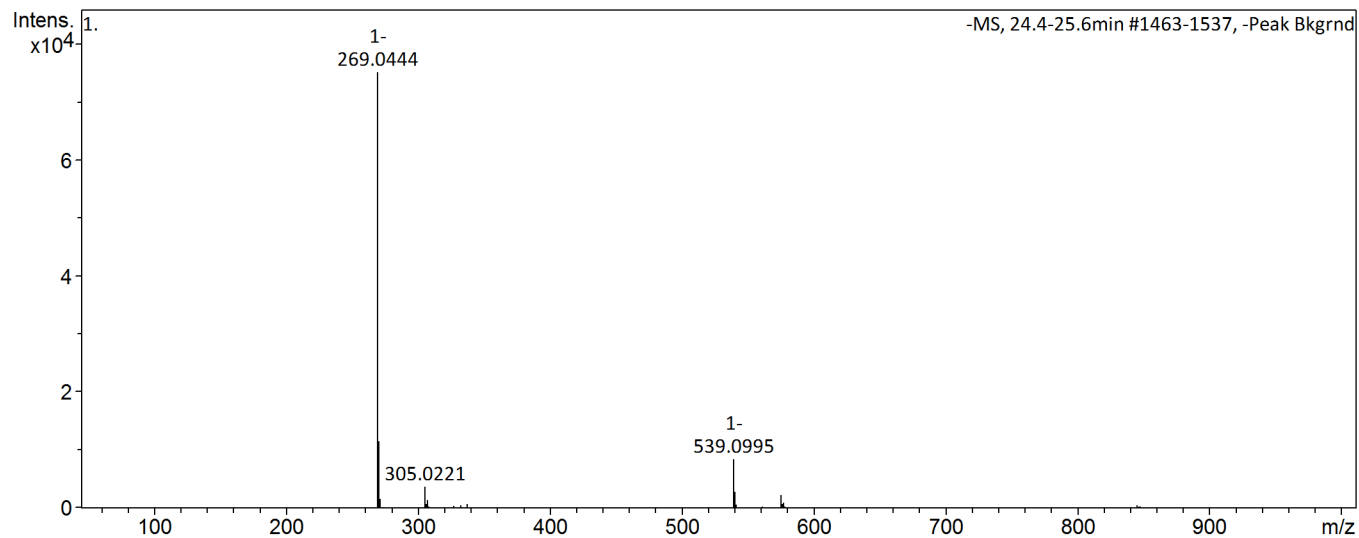

Supplement: Supplemental Information 3 [file peerj-09-12139-s003.pdf]

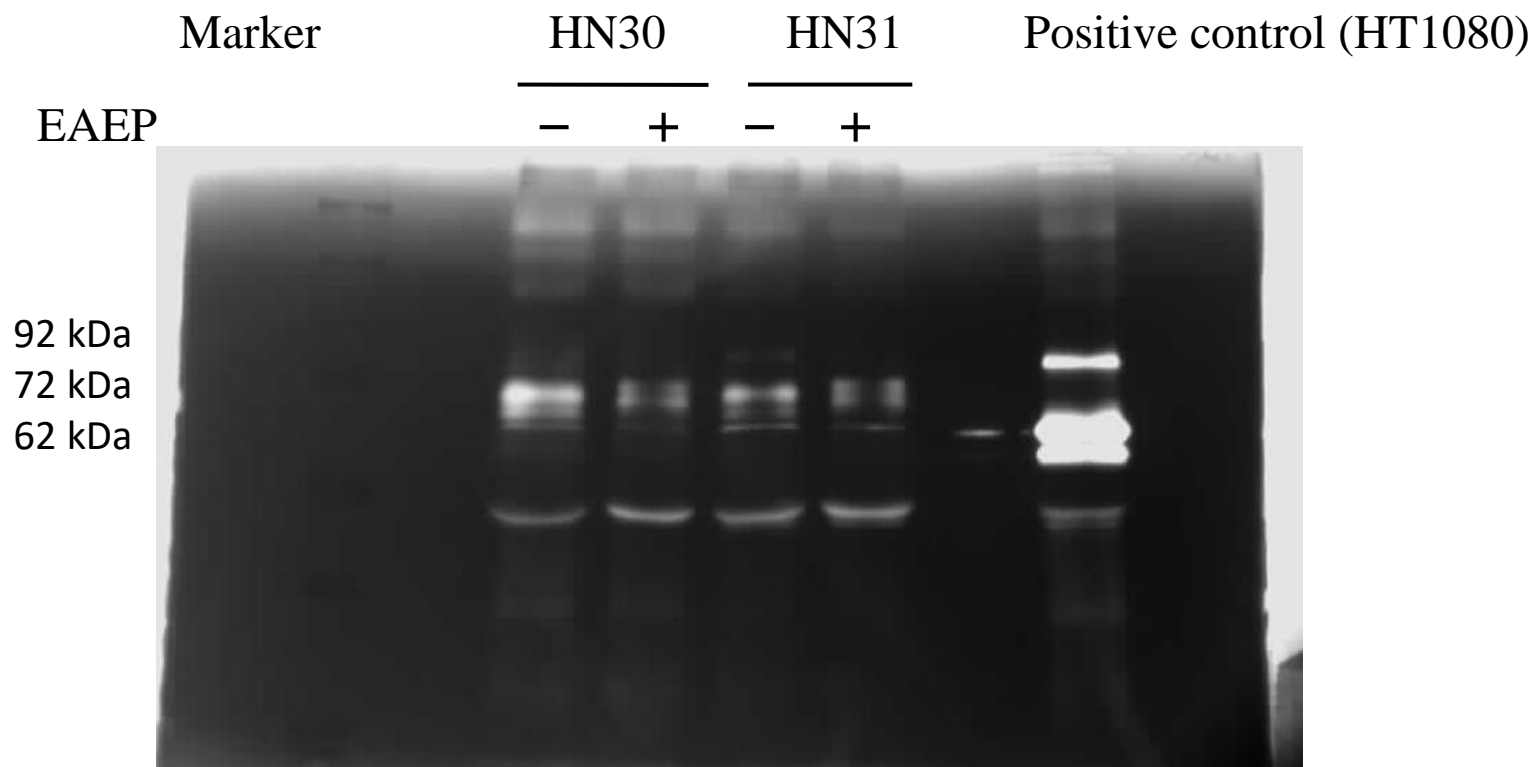

| Marker | HN4 |   | HN12 |   | Positive control (HT1080) |
|--------|-----|---|------|---|---------------------------|
| EAEP   | -   | + | -    | + |                           |

92 kDa  
72 kDa  
62 kDa

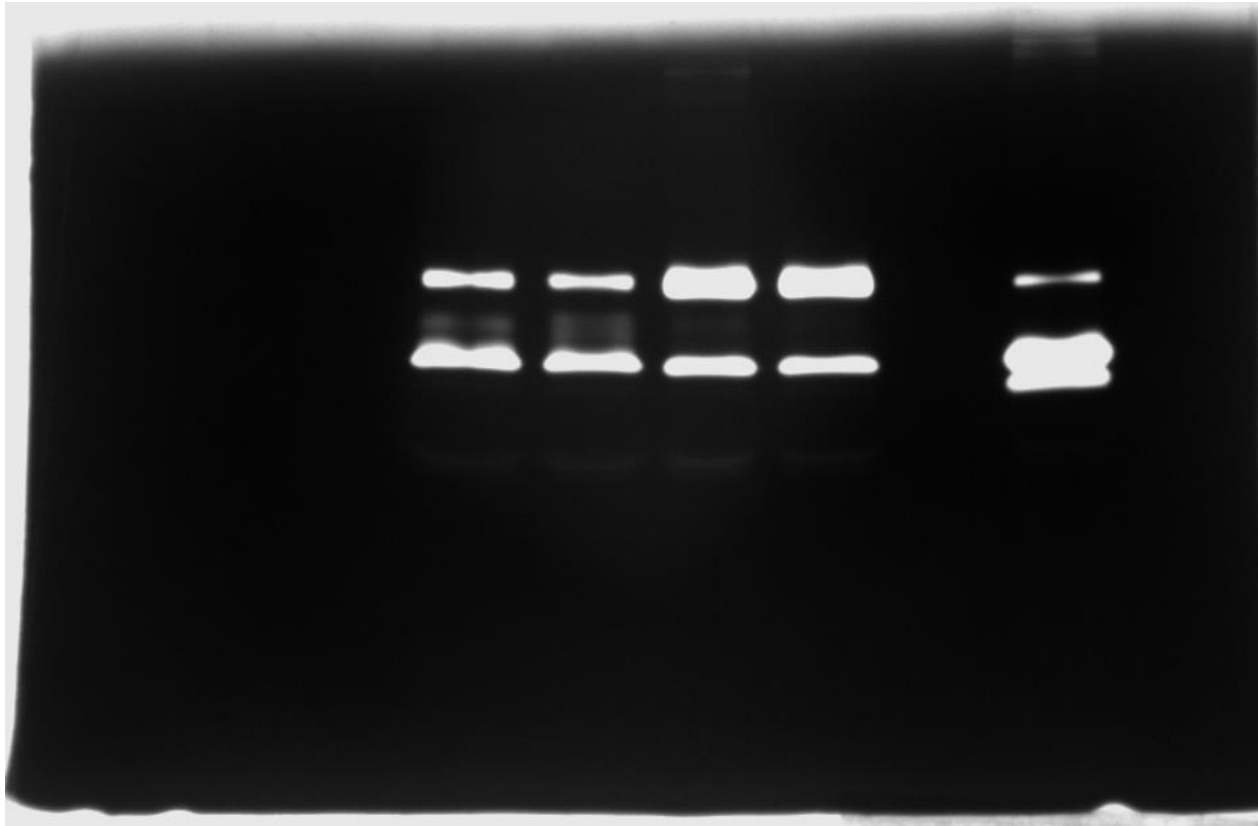

Supplement: Supplemental Information 5 — Zymographic gels for indicating MMPs activity in HN30, HN31, HN4 and HN12 cell lines. [file peerj-09-12139-s005.pdf]
